# Evaluation of morning bradykinesia in Parkinson’s disease in a United States cohort using continuous objective monitoring

**DOI:** 10.1016/j.prdoa.2022.100145

**Published:** 2022-05-17

**Authors:** S.H. Isaacson, R. Pahwa, E.J. Pappert, D. Torres-Russotto

**Affiliations:** a Parkinson's Disease and Movement Disorders Center of Boca Raton, 951 NW 13th St., Bldg. 5-E, Boca Raton, FL 33486, USA; b University of Kansas Medical Center, 3599 Rainbow Blvd, Kansas City, KS 66103, USA; c Sunovion Pharmaceuticals Inc., 84 Waterford Dr, Marlborough, MA 01752, USA; d University of Nebraska Medical Center, 42nd and Emile St, Omaha, NE 68198, USA

**Keywords:** Parkinson’s disease, Bradykinesia, Wearable devices, Continuous objective measurement, Morning OFF

## Abstract

**Introduction:**

Bradykinesia in Parkinson’s disease is a marker for clinical levodopa responsiveness, with persistent bradykinesia reflecting suboptimal response. We objectively measured prevalence and severity of morning bradykinesia using the Personal KinetiGraph® (PKG®).

**Methods:**

Retrospective evaluation of a large global database of de-identified PKG assessments from individuals (N=12,840) in routine clinical care in the United States (US; n=3288). Median bradykinesia scores (mBKS) and median dyskinesia scores (mDKS) were calculated using a validated algorithm and previously established targets to evaluate percent time in bradykinesia, levodopa responsiveness, and prevalence and severity (0–5; 5=highest severity) of morning bradykinesia.

**Results:**

mBKS was above target (≥26) in 65% of all individuals, and mDKS was above target (≥7) in 3%. Elevated percent time in bradykinesia occurred in 79%. Among individuals where levodopa responsiveness could be evaluated (n=1933), 31% had a significant response (≥1.15 postdose decrease in severity). Morning bradykinesia was identified in 85% of individuals with available morning data (1298/1524), and 64% (954/1501) experienced continued bradykinesia after the first daily levodopa dose. Morning bradykinesia was severe (4.0–4.7) in levodopa-responsive individuals regardless of percent time spent in bradykinesia.

**Conclusion:**

Elevated mBKS was very common in the US. Most individuals taking levodopa had morning bradykinesia that persisted even after the first daily dose, and severity was high, indicating a need for additional treatment options.

## Highlights

• The Personal KinetiGraph® detected morning bradykinesia in 85% of US individuals.
• After the first carbidopa/levodopa dose, 64% had continued morning bradykinesia.
• Morning bradykinesia was severe in levodopa-responsive individuals.
• Data suggest that some US individuals with PD may be undertreated or need additional treatment.

## 1 Introduction

Parkinson’s disease (PD) is a progressive neurodegenerative disorder with a global prevalence of approximately 1–2% in those ≥ 60 years of age [1]. Neurodegeneration in PD is widespread, resulting in myriad motor and nonmotor symptoms [1]. Degeneration of nigral dopaminergic neurons leads to striatal dopamine deficiency and the cardinal motor signs of bradykinesia, rest tremor, and rigidity [1].

Levodopa, administered with the dopa decarboxylase inhibitor carbidopa or benserazide, is the primary treatment for motor symptoms of PD and its beneficial effects are typically robust and consistent, especially in initial stages [1], [2]. Over time, patients taking levodopa may experience motor complications, including motor fluctuations and dyskinesia [3]. Motor fluctuations consist of periods when motor (including bradykinesia) and some nonmotor symptoms improve after a dose of levodopa (“ON”), followed by periods when the dose no longer provides benefit and symptoms recur (“OFF” episodes) [1], [4]. Approximately 50% of patients with PD develop “OFF” episodes after 5 years of levodopa treatment and 70% develop “OFF” episodes beyond 9 years [3].

Patients with PD can experience different types of “OFF” episodes, including morning “OFF.” Morning “OFF” is common throughout the course of PD, with an estimated prevalence of ∼60% [5]. Morning “OFF” consists of morning akinesia or bradykinesia after waking and before onset of effect of the first daily levodopa dose [4]. The cause of morning “OFF” may reflect a combination of loss of benefit from the last levodopa dose the night before or delayed onset of the first daily levodopa dose, reflecting delayed gastric emptying and pharmacodynamic effects [4]. Morning “OFF” episodes cause significant disability, which negatively impact health-related quality of life [6].

Timing, duration, frequency, and severity of “OFF” can sometimes be difficult to define clinically. Traditional methods of reporting “OFF” symptoms include self-reported patient diaries and questionnaires [7]. These methods are often used to describe clinical symptoms, are based on patient perception, and are subject to recall bias [7], [8]. Other difficulties associated with patient diaries have been well documented and include reduced compliance and diary fatigue [9]. Electronic patient diary usage has been mostly limited to clinical trials where patients are carefully screened based on their ability to complete these tools [9]. Anosognosia caused by PD or medications can lead to reduced awareness of bradykinesia and dyskinesia, making it difficult for patients to identify “OFF” and accurately relate “OFF” episode symptom severity, timing, and duration [10], [11], [12], [13], [14], [15].

PD symptom monitoring using a wearable continuous objective measurement (COM) device in the routine care setting is a recent development [16]. Objective measures reference “OFF” to a predefined score regardless of the patient’s perception of their level of bradykinesia [16]. The COM systems offer an opportunity to better detect “OFF” time along with time in bradykinesia and dyskinesia over an extended period and to monitor treatment responses [16]. A previous study comparing diaries and objective measurement showed that patients whose bradykinesia levels were consistently higher tended to identify “OFF” at higher objective scores [17]. Patient diaries categorize only 2 states of bradykinesia (“ON” or “OFF”) in a binary fashion [9]. Assessments such as the Unified Parkinson’s Disease Rating Scale (UPDRS) Part III are scalar, while COM devices like the Personal KinetiGraph® (PKG®; Global Kinetics Pty Ltd, Melbourne, Australia) provide a continuous bradykinesia range assessed during routine daily activities over a 6-day period [16]. Patients may alternate between target range (controlled PD symptoms) and out of target range (uncontrolled PD symptoms), and objective measurements provided by the PKG are an alternate method to characterize symptoms in order to optimize PD treatment [16].

The primary objective of our study was to use the PKG system to examine prevalence and severity of morning bradykinesia (a surrogate measure for morning “OFF”) in a United States (US) cohort.

## 2 Methods

### 2.1 The PKG system

The PKG system is a novel COM technology that consists of a wrist-worn watch, proprietary algorithms, and a data-driven report known as the PKG (Fig. S1). The PKG system, worn by patients with PD, delivers standardized continuous daily movement data on bradykinesia, dyskinesia, fluctuations, tremor, and immobility (proxy for daytime sleepiness), provides indicators of motor variations (eg, bradykinesia score [BKS] and dyskinesia score [DKS]), and indicates medication adherence via dose reminders [18], [19], [20], [21], [22]. The validated PKG algorithms [18], [19], [20], [21], [22] were designed to assess bradykinesia and dyskinesia, measure the effects of treatment, and to associate these with validated clinical rating scales (eg, UPDRS [23], Abnormal Involuntary Movement Scale [24], [25]). While there are no contraindications for the use of the PKG system, it is not intended to be used in patients who are nonambulatory, regulatory clearance in the US applies to patients who are 46–83 years of age, and the system has been used in patients with early- through late-stage PD.

## 3 Data source

The global PKG database was initiated in January 2012 and contains PKG data collected from countries where the PKG system was approved for use [26]. The data used in this study were taken from PKG assessments performed in routine clinical care of individuals with PD from US clinics that placed orders for the PKG system for their patients (2012–2019) and were collected during routine daily activities of the patient (usually over 6 days) [26]. Data from clinics with infrequent use of the PKG (≤45 individuals) were excluded. The first PKG from each individual was used in the analyses to control for a potentially biased group of patients dominating the dataset by appearing multiple times and to reduce treatment bias after the first PKG. The collection of demographic data was limited by the amount of information that individual clinic sites submitted within the PKG order form.

The PKG data capture system allows remotely captured patient data based on activities of daily living to be uploaded into larger regional or global databases and, once appropriately de-identified to remove patient health information, to be used for research [16]. The de-identified PKG database contains basic demographic data (eg, patient date of birth and sex, record number, clinic name, session duration and start date, reason for PKG, medications, and use of deep brain stimulation or infusion therapy), measures of bradykinesia, dyskinesia, percentage of time immobile, fluctuations and time in tremor, medication reminders to obtain peridose response, and number of days the PKG is worn by the patient. No identifiable protected health information was extracted, accessed, or used. Pursuant to the US Health Insurance Portability and Accountability Act of 1996 with updated provisions [27], this study used de-identified or anonymous data. Therefore, the study did not require Institutional Review Board or ethics committee approval or waiver of authorization.

### 3.1 Data analysis

A computational model was previously published that used PKG data from the first morning dose to predict absolute change in UPDRS Part III Motor Examination scores from a levodopa challenge test (area under the receiver operating characteristics curve, 0.92) [28]. Details relevant to the current study are described herein. First, the UPDRS Part III score was divided into 6 “severity” levels (0–5; 5=highest severity) so that motor function severity could be categorically classified (Table 1). Second, an algorithm was developed to categorize bradykinesia severity into 6 different levels in 2-minute recordings (ie, epochs) between 09:00 and 18:00. The algorithm was based on a logistic regression model of UPDRS Part III scores and allowed for measurement of the proportion of time an individual spent in bradykinesia and their levodopa response.

**Table 1:** Bradykinesia severity levels and association with UPDRS Part III scores.

Severity Level^a^	0	1	2	3	4	5
UPDRS Part III Interval	0 to <10	≥10 to <22.5	≥22.5 to <35	≥35 to <47.5	≥47.5 to <60	≥60

UPDRS, Unified Parkinson’s Disease Rating Scale.

a Severity levels as per Khodakarami et al [26].

Bradykinesia was defined as “in target” and “controlled” when an epoch was categorized with a severity level <2.5 and “above target” and “uncontrolled” (ie, “OFF”) with a severity level ≥2.5. A severity level of 2.5 was equivalent to a UPDRS Part III score of 35, ∼55% of time spent in bradykinesia, and ∼3 hours of “OFF” time from 09:00 to 18:00.

Epochs were excluded from analysis if the BKS was ≥40, which is indicative of inactivity (BKS 40 to <80) or sleep (BKS ≥80) [22], [29], if the watch was sensed as not being worn, or if there was a standard deviation of >1 severity level for levodopa responsiveness 5 epochs (10 min) after the first levodopa dose reminder or ∼46–90 min after the time of first dose administration.

## 4 Assessments and definitions

For each epoch the PKG watch was worn, a BKS and DKS was produced. Median BKS (mBKS) and median DKS (mDKS) was defined as the 50th percentile of BKS and DKS, respectively, and was calculated for all days the PKG was worn (minimum of 5 days but usually 6 days) [19]. Target therapeutic ranges for mBKS and mDKS were <26 and <7, respectively. Any score above these thresholds was considered abnormal (ie, “in bradykinesia” or “in dyskinesia”) [30].

Objective measure score of percent time in bradykinesia combines the concept of being “in bradykinesia” (ie, “OFF” by diaries) and ranges of UPDRS Part III score “severity” described above. Percent time in bradykinesia was estimated as the percentage of time that individuals spent in severity levels >2.5 (ie, levels 3–5 [UPDRS Part III score >35]) between 09:00 and 18:00 during the 6 days the PKG watch was worn, expressed as a percentage of all available epochs in that period [28]. Upper limit of the percent time in bradykinesia for control individuals was 30% [30].

Levodopa responsiveness was estimated by calculating improvement in BKS at effect time versus time of the first daily levodopa dose. A significant levodopa response corresponded with a severity level improvement (ie, decrease) ≥1.15 units [28], (∼14-point reduction in UPDRS Part III score) after the first daily levodopa dose. Levodopa responsiveness was further classified as fluctuators versus nonfluctuators based on epochs collected around the time of the first daily levodopa dose to determine the proportion with morning bradykinesia (ie, uncontrolled nonfluctuators and fluctuators with respect to their levodopa response). Uncontrolled nonfluctuators were defined as individuals whose bradykinesia was above target (mBKS ≥26; Table 1) at time of the first daily levodopa dose and remained above target after the first dose without evidence of a significant levodopa response (Fig. S2). Fluctuators were individuals whose bradykinesia was above target at time of the first daily levodopa dose and who experienced a significant levodopa response. Controlled nonfluctuators were individuals whose bradykinesia was in target at the time of the first daily levodopa dose (ie, no morning bradykinesia) and whose scores remained in target after the first dose.

## 5 Results

### 5.1 Bradykinesia and dyskinesia at a population level

The mBKS and mDKS from 3288 individuals with PD in the US were included in the analysis. Males represented 67% of the population and the median age was 71.9 years. mBKS was 28.6 and bradykinesia was above target (mBKS ≥26) in 65% of individuals. The mDKS was 1.1 and dyskinesia was above target (mDKS ≥7) in 3% of individuals.

### 5.2 Percent time in bradykinesia and levodopa responsiveness

**Fig 1:**
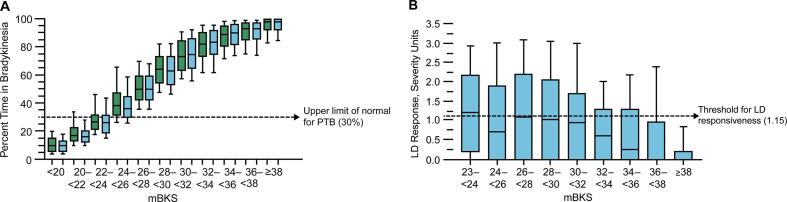
Comparisons of (A) PTB and (B) LD responsiveness with mBKS. Horizontal line in the boxes represents the median, and whiskers denote the 10th and 90th percentiles. Green boxes denote all individuals. Blue boxes denote those individuals with data for LD responsiveness. LD, levodopa; mBKS, median bradykinesia score; PTB, percent time in bradykinesia. (For interpretation of the references to colour in this figure legend, the reader is referred to the web version of this article.)

Percent time in bradykinesia increased with increased mBKS (Fig. 1A). Elevated percent time in bradykinesia (≥30%) was estimated to have occurred in 79% of individuals with PD, including all individuals with mBKS ≥26, most individuals with mBKS 24 to <26, and in a small proportion of individuals with mBKS 22 to <24 (Fig. 1A).

Data for evaluation of levodopa responsiveness were available in 59% (n=1933) of individuals, and the distribution of mBKS against percent time in bradykinesia was similar to the distribution for all US individuals (Fig. 1A). Significant levodopa response (severity level improvement ≥1.15 units after the first daily levodopa dose) was observed in approximately one-third of individuals with evaluable data (n=677; Fig. 1B). Significant levodopa responses became progressively less common with increased mBKS; 39–50% with lower mBKS (23 to <28), 30–46% in the middle mBKS groups (28 to <34), and 8–29% with higher mBKS (34 to ≥38; Fig. 1B).

### 5.3 Morning bradykinesia

**Fig. 2:**
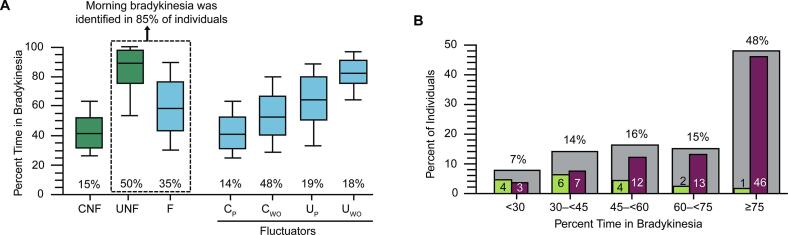
Evaluation of (A) PTB by classification of levodopa responsiveness and (B) percent of individuals with bradykinesia from 09:00 to 18:00 by PTB grouping with evaluable morning data. Panel A shows the PTB of F, CNF, and UNF and subtypes of F (C_P_ C_WO_, U_P_, and U_WO_). Percentages along the x-axis show their relative proportions. CNF: an individual whose bradykinesia was controlled (mBKS <26) at time of the first daily levodopa dose and remained controlled after the first dose. F: an individual whose bradykinesia was above target (mBKS ≥26) at time of the first daily levodopa dose and who experienced a significant levodopa response. UNF: an individual whose bradykinesia was above target at time of the first daily levodopa dose and remained above target after the first dose without evidence of a significant levodopa response. C_P_: an individual whose bradykinesia is below target after the first daily levodopa dose and the bradykinesia persists below target for>2 h after the peak response. C_WO_: an individual whose bradykinesia is below target after the first daily levodopa dose and subsequently experiences wearing “OFF.” U_P_: an individual whose bradykinesia is above target after the first daily levodopa dose and persists. U_WO_: an individual whose bradykinesia is above target after the first daily levodopa dose and who experiences a wearing “OFF” of the levodopa response they did experience. Panel B: Gray bars show the relative percentage of individuals with evaluable morning data experiencing bradykinesia from 09:00 to 18:00 in each PTB range, while green bars and maroon bars show the percentage with morning bradykinesia that is below or above target, respectively. Numbers may not sum to 100% due to rounding. CNF, controlled nonfluctuator; C_P_, controlled persisting; C_WO_, controlled wearing “OFF”; F, fluctuator; mBKS, median bradykinesia score; PTB, percent time in bradykinesia; UNF, uncontrolled nonfluctuator; U_P_, uncontrolled persisting; U_WO_, uncontrolled wearing “OFF.” (For interpretation of the references to colour in this figure legend, the reader is referred to the web version of this article.)

Of 1933 individuals with data to evaluate levodopa responsiveness, 1524 had evaluable data for morning bradykinesia. Morning bradykinesia was identified in 85% (n=1298) of individuals, inclusive of 50% (n=761) who were uncontrolled nonfluctuators and 35% (n=537) who were fluctuators (Fig. 2A). Median values for percent time in bradykinesia were elevated in all 3 levodopa responsiveness groupings (uncontrolled nonfluctuators, fluctuators, and controlled nonfluctuators); the highest median value was observed in uncontrolled nonfluctuators. Data for subclassification of fluctuators were available in 514 of 537 individuals, and 48% (n=249) had bradykinesia within target therapeutic range after their first daily levodopa dose, but later experienced time above target (Fig. 2A). Sixty-four percent (954/1501) of individuals experienced continued morning bradykinesia after the first daily levodopa dose.

A total of 48% of individuals (725/1524) with evaluable morning bradykinesia data spent ≥75% of their time in bradykinesia from 09:00 to 18:00 (Fig. 2B). Of individuals who exhibited a significant levodopa response, the severity level of morning bradykinesia was high (4.0–4.7 on a scale of 0–5) regardless of percent time in bradykinesia (Table 2). Severity level of morning bradykinesia was <2 in individuals with an insignificant levodopa response.

**Table 2:** Severity level of morning bradykinesia by percent time in bradykinesia grouping in individuals with insignificant and significant LD responsiveness in the US cohort.

	**Severity Level of Morning Bradykinesia,**^a^ **Mean (SD)**	
**Percent Time in Bradykinesia Grouping**	**Individuals With Significant LD Response**^b^	**Individuals With Insignificant LD Response**^b^
<30 (n=111)	4.0 (0.9)	1.5 (0.6)
30–<45 (n=211)	4.5 (0.7)	1.5 (0.5)
45–<60 (n=244)	4.1 (0.9)	1.6 (0.6)
60–<75 (n=233)	4.3 (0.8)	1.8 (0.5)
≥75 (n=725)	4.7 (0.5)	1.8 (0.5)

LD, levodopa; SD, standard deviation; US, United States.

a Severity levels as per Table 1.

b A significant levodopa response corresponded with a severity level improvement (ie, decrease) of ≥ 1.15 units (see Table 1) after the carbidopa/levodopa dose.

## 6 Discussion

Levodopa is the cornerstone treatment for motor symptoms of PD, but the pharmacologic response inevitably decreases over time [1]. Initial approaches to manage patients who no longer have an adequate levodopa response include optimizing levodopa treatment (eg, increasing doses, altering dosing frequency or formulations, etc) and adding “ON-extenders” (eg, dopamine agonists, monoamine oxidase-B inhibitors, etc) [2], but symptom control may still be suboptimal and elusive. In this study of individuals with PD in the US receiving routine clinical care, mBKS as measured by the PKG system was observed to be above target in approximately two-thirds of individuals evaluated, and mBKS was 2 points higher than the target, which approximates to ∼3–4 points on the UPDRS Part III score. Elevated percent time in bradykinesia was estimated to have occurred in >75% of individuals with PD. While it was not surprising to observe that individuals with mBKS above target therapeutic range (≥26) for antiparkinsonian treatment were within range for elevated percent time in bradykinesia, it is noteworthy that some individuals with mBKS scores below target mBKS (22 to <26) also had elevated percent time in bradykinesia. This would indicate that some patients may suffer from clinically relevant bradykinesia or “OFF” time that requires optimization of existing or additional treatment despite a controlled mBKS.

The levodopa challenge test cannot always be estimated from the PKG. It requires dose reminders to be set indicating time of the first daily dose and for there to be sufficient data at time of the first daily dose and at peak response. The latter may not occur if individuals were asleep or did not wear the PKG watch at that time. We estimated the levodopa challenge test in 59% of individuals, and a significant levodopa response was observed in only ∼1 out of 3 individuals. Not surprisingly, the proportion of individuals who had significant levodopa response decreased with increased mBKS grouping and the trend was most noticeable starting with individuals with mBKS above target (≥26).

Morning “OFF” is highly prevalent in patients with PD who are treated with levodopa, and time to “ON” for a morning levodopa dose may be ≥60 min [31]. In a multicenter, observational study by Rizos et al, the prevalence of morning “OFF” was estimated to be 60% in 320 patients with PD [5]. In that study, the presence of morning “OFF” was identified through a combination of structured interview questions and responses to validated instruments (eg, UPDRS, Parkinson’s Disease Sleep Scale). More recently, Stocchi et al estimated the prevalence of delayed “ON,” a component of morning “OFF,” at 51% in a single-visit pilot study of 151 patients with PD [32]. Delayed “ON” in this study was identified from a custom questionnaire. Notably, using measurement of continuous movement from the PKG system in our study, morning bradykinesia was observed in ∼25–35% more individuals than observed in the aforementioned studies where more conventional methods were employed. This possibly suggests that morning “OFF” may be underdetected or underdiagnosed and that the PKG system could help to supplement clinician-directed patient interviews and validated assessment tools. At least one prior study of the PKG system, inclusive of 1752 hours of data collected from 24 patients with PD, has confirmed significant correlation of COM data to data obtained by conventional diaries for bradykinesia while “OFF” [17].

The finding that morning bradykinesia persisted in approximately two-thirds of individuals after the first daily levodopa dose, and that approximately half of those with morning bradykinesia spent ≥75% of their daytime hours in bradykinesia, points to a striking level of possible undertreatment in the US. Undertreatment in this context does not mean levodopa responsiveness is absent; rather, that the dose level of levodopa may not be sufficiently high. The strategy for PD treatment should be to lower bradykinesia without increasing dyskinesia. Therefore, the PKG system could be another tool to help clinicians identify patients who may benefit from optimization of current treatments or addition of new “ON-extenders,” and for monitoring responsiveness to treatment after regimens are modified, including possible dyskinesia.

A strength of the study includes the large sample size of individuals in routine clinical practice that was analyzed. As with all COM technology, observed data may be limited by artifacts or the real-world conditions in which individuals were using the recording device. For example, it is possible that some individuals were inactive or took some of their regularly scheduled PD medications within 8 hours of the levodopa challenge test, whereas ideal circumstances call for patients to be out of bed, active, and having taken no PD medications within 8 hours. While data were collected from clinics with frequent use of the PKG (use in >45 individuals), there was no limit on the type of clinic (general neurology clinic, movement disorder clinic, etc) that could employ the PKG and there was potential for inclusion of individuals other than those with idiopathic PD in the data set. In addition, information on the dosage of levodopa and other concomitant medications (for PD and non-PD conditions) were not collected. Therefore, estimates of bradykinesia prevalence, reduced levodopa responsiveness, or possible undertreatment could be further refined in future research.

## 7 Conclusion

Data obtained from COM technology using the PKG system suggest that a substantial number of individuals with PD in the US treated with levodopa have morning bradykinesia, which persists in most individuals after the first daily dose. Among individuals with morning bradykinesia, approximately half spent most of their time in bradykinesia during the day, and the severity level of morning bradykinesia was high. Many patients in the US may experience a limited beneficial response to their first daily levodopa dose. This may reflect dopaminergic undertreatment, which can benefit from optimizing their current treatment regimen or addition of “ON-extenders.”

## Author's roles

All authors (SHI, RP, EJP, DT-R) contributed to the conception and design and have been involved in drafting the manuscript and revising it for critically important intellectual content. All authors read and approved the final manuscript and agree to be accountable for all aspects of the work.

## Funding

This study and preparation of the manuscript was supported by funding from Sunovion Pharmaceuticals Inc. (Marlborough, Massachusetts, USA). The funding body did not participate in study design; in the collection, analysis, and interpretation of data; in the writing of the report; and in the decision to submit the article for publication.

**Prior Publication:** Presented in part at the 2020 American Academy of Neurology (AAN) Science Highlights Virtual Platform and the Virtual International Association of Parkinsonism and Related Disorders (IAPRD) XXVI World Congress.

## Declaration of Competing Interest

SHI, RP, and DT-R are consultants for Sunovion Pharmaceuticals Inc. (Marlborough, MA, USA) and Global Kinetics Pty Ltd (Melbourne, Australia). EJP is an employee of Sunovion Pharmaceuticals Inc.

SHI reports honoraria for CME, consultant, research grants, and/or promotional speaker on behalf of AbbVie, Acadia Pharmaceuticals Inc, Acorda Therapeutics, Inc., Adamas Pharmaceuticals, Inc., Addex Therapeutics, AFFiRiS AG, Alexza Pharmaceuticals, Allergan, Amarantus BioScience, Amneal Pharmaceuticals, Aptinyx, Axial Therapeutics, Inc., Axovant Gene Therapies, BenevolentAI, Biogen, Britannia Pharmaceuticals, Cadent Therapeutics, Cala Health, Cerecor, Inc., Cerevel Therapeutics, Cipla, Eli Lilly, Enterin Inc., GE Healthcare, Global Kinetics Pty Ltd, Impax Laboratories, Impel NeuroPharma, Intec Pharma, Ipsen, Jazz Pharmaceuticals, Kyowa Kirin, Lundbeck, Merz Pharmaceuticals, Michael J. Fox Foundation, Mitsubishi Tanabe Pharma, Neuraly, Neurocrine Biosciences, NeuroDerm, Parkinson Study Group, Pharma Two B, Prilenia Therapeutics, Promentis Pharmaceuticals, Inc., Revance, Roche, Sanofi, Sunovion Pharmaceuticals Inc., Sun Pharma, Supernus Pharmaceuticals, Inc., Teva, Theravance Biopharma, and UCB.

RP reports honoraria, consultant fees, and/or research grants from Abbott Laboratories, AbbVie, Acadia Pharmaceuticals Inc, Acorda Therapeutics Inc., Adamas Pharmaceuticals, Inc., Amneal Pharmaceuticals, Biogen, Biohaven Pharmaceuticals Inc., Boston Scientific, Cala Health, EIP Pharma Inc., Eli Lilly, Global Kinetics Pty Ltd, Kyowa Kirin, Lundbeck, Mitsubishi Tanabe Pharma, Neuraly, Neurocrine Biosciences, Parkinson Foundation, Pharma Two B, Prelinia Therapeutics, Roche, Sage Therapeutics, Sun Pharma, Sunovion Pharmaceuticals Inc., Theranexus, Theravance, US WorldMeds, and Voyager.

DT-R reports honoraria, consultant, research grants, and/or promotional speaker on behalf of AbbVie, Acadia Pharmaceuticals Inc, Acorda Therapeutics Inc., Adamas Pharmaceuticals, Inc., Global Kinetics Pty Ltd, Ipsen, Revance, Sunovion Pharmaceuticals Inc., and Teva.

## References

[1] Olanow C.W., Stern M.B., Sethi K. (2009). The scientific and clinical basis for the treatment of Parkinson disease (2009). Neurology.

[2] Armstrong M.J., Okun M.S. (2020). Diagnosis and treatment of Parkinson disease: a review. JAMA.

[3] Ahlskog J.E., Muenter M.D. (2001). Frequency of levodopa-related dyskinesias and motor fluctuations as estimated from the cumulative literature. Mov. Disord..

[4] Chou K.L., Stacy M., Simuni T., Miyasaki J., Oertel W.H., Sethi K., Fernandez H.H., Stocchi F. (2018). The spectrum of “off” in Parkinson's disease: what have we learned over 40 years?. Parkinsonism Relat. Disord..

[5] Rizos A., Martinez-Martin P., Odin P., Antonini A., Kessel B., Kozul T.K., Todorova A., Douiri A., Martin A., Stocchi F., Dietrichs E., Chaudhuri K.R. (2014). Characterizing motor and non-motor aspects of early-morning off periods in Parkinson's disease: an international multicenter study. Parkinsonism Relat. Disord..

[6] Chapuis S., Ouchchane L., Metz O., Gerbaud L., Durif F. (2005). Impact of the motor complications of Parkinson's disease on the quality of life. Mov. Disord..

[7] Stone A.A., Shiffman S. (2002). Capturing momentary, self-report data: a proposal for reporting guidelines. Ann. Behav. Med..

[8] Stone A.A., Shiffman S., Schwartz J.E., Broderick J.E., Hufford M.R. (2002). Patient non-compliance with paper diaries. BMJ.

[9] Papapetropoulos S.S. (2012). Patient diaries as a clinical endpoint in Parkinson's disease clinical trials. CNS Neurosci. Ther..

[10] Maier F., Prigatano G.P. (2017). Impaired self-awareness of motor disturbances in Parkinson's disease. Arch. Clin. Neuropsychol..

[11] Matthews H., Stamford J., Saha R., Martin A. (2015). Exploring issues around wearing-off and quality of life: the OFF-PARK Survey of people with Parkinson's disease and their care partners. J. Parkinsons. Dis..

[12] Pietracupa S., Fasano A., Fabbrini G., Sarchioto M., Bloise M., Latorre A., Altieri M., Bologna M., Berardelli A. (2013). Poor self-awareness of levodopa-induced dyskinesias in Parkinson's disease: clinical features and mechanisms. Parkinsonism Relat. Disord..

[13] Maier F., Ellereit A.L., Eggers C., Lewis C.J., Pelzer E.A., Kalbe E., Ernstmann N., Prigatano G.P., Fink G.R., Timmermann L. (2015). Development and psychometric evaluation of a scale to measure impaired self-awareness of hyper- and hypokinetic movements in Parkinson's disease. J. Int. Neuropsychol. Soc..

[14] Maier F., Prigatano G.P., Kalbe E., Barbe M.T., Eggers C., Lewis C.J., Burns R.S., Morrone-Strupinsky J., Moguel-Cobos G., Fink G.R., Timmermann L. (2012). Impaired self-awareness of motor deficits in Parkinson's disease: association with motor asymmetry and motor phenotypes. Mov. Disord..

[15] Pennington C., Duncan G., Ritchie C. (2020). Altered awareness of motor symptoms in Parkinson's disease and Dementia with Lewy Bodies: a systematic review. Int. J. Geriatr. Psychiatry.

[16] Pahwa R., Isaacson S.H., Torres-Russotto D., Nahab F.B., Lynch P.M., Kotschet K.E. (2018). Role of the Personal KinetiGraph in the routine clinical assessment of Parkinson's disease: recommendations from an expert panel. Expert Rev. Neurother..

[17] Ossig C., Gandor F., Fauser M., Bosredon C., Churilov L., Reichmann H., Horne M.K., Ebersbach G., Storch A. (2016). Correlation of quantitative motor state assessment using a Kinetograph and patient diaries in advanced PD: data from an observational study. PLoS ONE.

[18] Braybrook M., O'Connor S., Churchward P., Perera T., Farzanehfar P., Horne M. (2016). An ambulatory tremor score for Parkinson's disease. J. Parkinsons Dis..

[19] Griffiths R.I., Kotschet K., Arfon S., Xu Z.M., Johnson W., Drago J., Evans A., Kempster P., Raghav S., Horne M.K. (2012). Automated assessment of bradykinesia and dyskinesia in Parkinson's disease. J. Parkinsons Dis..

[20] Horne M., Kotschet K., McGregor S. (2016). The clinical validation of objective measurement of movement in Parkinson’s disease. Oruen - CNS J..

[21] Horne M.K., McGregor S., Bergquist F. (2015). An objective fluctuation score for Parkinson's disease. PLoS ONE.

[22] Kotschet K., Johnson W., McGregor S., Kettlewell J., Kyoong A., O'Driscoll D.M., Turton A.R., Griffiths R.I., Horne M.K. (2014). Daytime sleep in Parkinson's disease measured by episodes of immobility. Parkinsonism Relat. Disord..

[23] Martinez-Martin P., Gil-Nagel A., Gracia L.M., Gomez J.B., Martinez-Sarries J., Bermejo F. (1994). Unified Parkinson's Disease Rating Scale characteristics and structure. The Cooperative Multicentric Group. Mov. Disord..

[24] Hughes A.J., Frankel J.P., Kempster P.A., Stern G.M., Lees A.J. (1994). Motor response to levodopa in patients with parkinsonian motor fluctuations: a follow-up study over three years. J. Neurol. Neurosurg. Psychiatry.

[25] Marsden C.D., Schachter M. (1981). Assessment of extrapyramidal disorders. Br. J. Clin. Pharmacol..

[26] Pahwa R., Bergquist F., Horne M., Minshall M.E. (2020). Objective measurement in Parkinson's disease: a descriptive analysis of Parkinson's symptom scores from a large population of patients across the world using the Personal KinetiGraph(R). J. Clin. Mov. Disord..

[27] Centers for Medicare & Medicaid Services, CFR-Title 45-Chapter A-Subchapter C-Part 164-Subpart E-Section 164.502, Subsection (d) Standard Uses and disclosures of de-identified protected health information. December 3, 2002; 45 CFR 164.502(d), and 164.514(a)-(c), 2002. https://www.law.cornell.edu/cfr/text/45/164.502.

[28] Khodakarami H., Ricciardi L., Contarino M.F., Pahwa R., Lyons K.E., Geraedts V.J., Morgante F., Leake A., Paviour D., De Angelis A., Horne M. (2019). Prediction of the levodopa challenge test in Parkinson's disease using data from a wrist-worn sensor. Sensors (Basel).

[29] McGregor S., Churchward P., Soja K., O'Driscoll D., Braybrook M., Khodakarami H., Evans A., Farzanehfar P., Hamilton G., Horne M. (2018). The use of accelerometry as a tool to measure disturbed nocturnal sleep in Parkinson's disease. NPJ Parkinsons Dis..

[30] Khodakarami H., Shokouhi N., Horne M. (2021). A method for measuring time spent in bradykinesia and dyskinesia in people with Parkinson's disease using an ambulatory monitor. J. NeuroEng. Rehabil..

[31] Isaacson S., Lew M., Ondo W., Hubble J., Clinch T., Pagan F. (2017). Apomorphine subcutaneous injection for the management of morning akinesia in Parkinson's disease. Mov. Disord. Clin. Pract..

[32] Stocchi F., Coletti C., Bonassi S., Radicati F.G., Vacca L. (2019). Early-morning OFF and levodopa dose failures in patients with Parkinson's disease attending a routine clinical appointment using Time-to-ON Questionnaire. Eur. J. Neurol..

