# Supplementary material for: Evaluation of morning bradykinesia in Parkinson’s disease in a United States cohort using continuous objective monitoring
Source: Clin Park Relat Disord. 2022 May 17;6:100145. doi: 10.1016/j.prdoa.2022.100145 (PMC9127405; doi:10.1016/j.prdoa.2022.100145)
Supplement: Supplementary data 1 [file mmc1.docx]

**Supplemental Materials**

Fig. S1. PKG System.


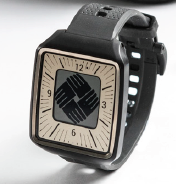


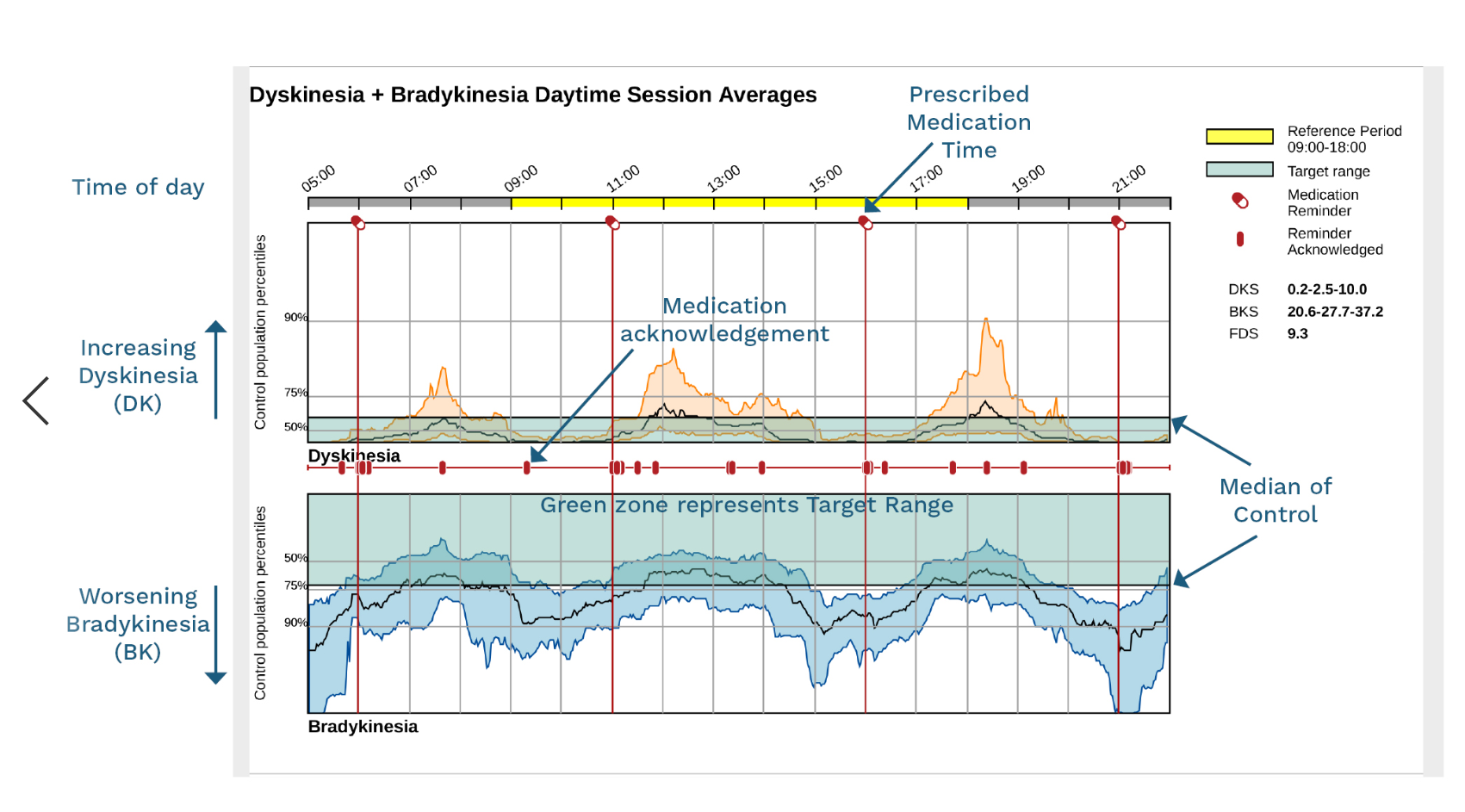


Top panel: PKG^®^ watch. Bottom panel: graphical representation of the data output (the PKG^®^) collected every 2 minutes over an extended period (typically 6 days). Bradykinesia is plotted against the time of day and the time when medications are due for administration. Image credits: Global Kinetics Pty Ltd.

BKS, bradykinesia score; DKS, dyskinesia score; FDS, fluctuation and dyskinesia score; PKG^®^, Personal KinetiGraph^®^.

Fig. S2. Classification of levodopa responsiveness.


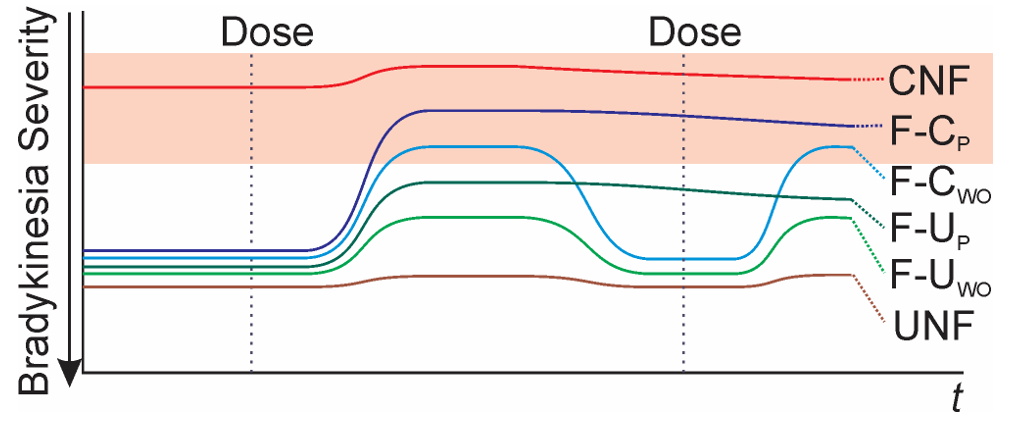


CNF, controlled nonfluctuator; C_P_, controlled persisting; C_WO_, controlled wearing “OFF”; F, fluctuator; t, time; UNF, uncontrolled nonfluctuator; U_P_, uncontrolled persisting; U_WO_, uncontrolled wearing “OFF.”
